# Critical comparison of American and European classifications of müllerian anomalies: pros and cons

**DOI:** 10.1590/0100-3984.2024.0096-en

**Published:** 2025-05-21

**Authors:** Luís Ronan Marquez Ferreira de Souza, Cinthia Callegari Barbisan, Cecília Vidal de Souza Torres, Isadora Balderama Canedo

**Affiliations:** 1 Universidade Federal do Triângulo Mineiro (UFTM), Uberaba, MG, Brazil; 2 Beneficência Portuguesa de São Paulo (BP), São Paulo, SP, Brazil; 3 Hospital das Clínicas da Faculdade de Medicina de Ribeirão Preto da Universidade de São Paulo (HCFMRP-USP), Ribeirão Preto, SP, Brazil

**Keywords:** Congenital abnormalities, Mullerian ducts, Classification, Diagnosis, Radiology, Diagnostic imaging, Anomalias congênitas, Ductos müllerianos, Classificações, Diagnóstico, Radiologia, Diagnóstico por imagem

## Abstract

Müllerian anomalies represent a spectrum of congenital malformations of the female reproductive tract. Over the decades, various classifications have been developed to categorize these anomalies. Based on a classification proposed by Kaufmann and Jarcho in 1946, the classification devised by the American Fertility Society in 1988 was considered simple and practical; although it faced criticism for its subjectivity and limitations in classifying complex anomalies, it was widely adopted. In 2013, the European Society of Human Reproduction and Embryology and the European Society for Gynaecological Endoscopy introduced a more detailed classification, which, albeit more complex and with a risk of overdiagnosis, also included cervical and vaginal anomalies. In 2021, the American Society for Reproductive Medicine updated the classification with the aim of simplifying and improving diagnostic accuracy, expanding the categories, and defining more objective criteria. This new classification seeks to facilitate communication among professionals and enhance clinical management, emphasizing the importance of continuous updates to improve reproductive outcomes and the quality of life for patients affected by these anomalies. This article aims to discuss the strengths and limitations of each of these classifications, offering a critical analysis of their impact on the diagnosis and treatment of müllerian anomalies. It also seeks to highlight aspects that may be refined in future revisions to achieve greater diagnostic precision and clinical applicability.

## Abstract (pt)

As anomalias müllerianas representam um espectro de malformações congênitas do trato reprodutivo feminino. Ao longo das décadas, diversas classificações foram criadas para categorizá-las. Inicialmente proposta por Kaufmann e Jarcho em 1946, a classificação da Sociedade Americana de Fertilidade de 1988 foi considerada simples e prática e foi amplamente utilizada, mas criticada por sua subjetividade e limitação em classificar anomalias complexas. Em 2013, a Sociedade Europeia de Reprodução Humana e Embriologia/Sociedade Europeia de Endoscopia Ginecológica introduziram uma classificação mais detalhada, incluindo também anomalias cervicais e vaginais, embora mais complexa e com risco de diagnósticos excessivos. A Sociedade Americana de Medicina Reprodutiva, em 2021, atualizou a classificação com o intuito de simplificar e melhorar a precisão diagnóstica, expandindo categorias e definindo critérios mais objetivos. A nova classificação visa facilitar a comunicação entre profissionais e aprimorar o manejo clínico, destacando a importância de atualizações contínuas para melhorar os resultados reprodutivos e a qualidade de vida das pacientes afetadas por essas anomalias. Este artigo tem como objetivo discutir os pontos fortes e as limitações de cada uma dessas classificações, fornecendo uma análise crítica sobre seus impactos no diagnóstico e tratamento das anomalias müllerianas. Além disso, propõe-se levantar aspectos que podem ser aprimorados em futuras revisões, visando maior precisão diagnóstica e aplicabilidade clínica.

## INTRODUCTION

Müllerian anomalies have a wide spectrum of presentations, and the complete categorization of these anomalies is therefore challenging. In recent decades, various professional societies have proposed classifications in order to identify the best diagnostic approaches.

The prevalence of müllerian anomalies varies considerably depending on the population studied, being up to 25% among women with a history of infertility and spontaneous abortions^**(^1^)**^. Such anomalies are associated with a higher incidence of premature births, as well as with premature rupture of the fetal membranes, fetal malpresentation, and perinatal mortality. The incidence of premature birth varies according to the type of anomaly, being higher in cases of uterus didelphys, whereas the risk of abortion is higher in women with a septate uterus^**(^2^)**^.

In this review of the literature, we compare the müllerian anomaly classifications proposed by the American Fertility Society (AFS) in 1979 and 1988^**(^3^,^4^)**^ with that proposed in 2021 by the American Society for Reproductive Medicine (ASRM) and with that proposed by the European Society of Human Reproduction and Embryology and the European Society for Gynaecological Endoscopy (ESHRE/ESGE) in 2013^**(^5^)**^. We highlight the advantages, limitations, and clinical implications of each classification, in order to contextualize their use in daily radiology practice.

## EMBRYOLOGY AND ANATOMY

The development of the female reproductive tract involves the differentiation of the müllerian ducts, driven by the absence of anti-müllerian hormone and by the activity of estrogen, resulting in the formation of the uterus, fallopian tubes, cervix, and upper vagina^**(^6^,^7^)**^. This process occurs in three stages^**(^8^)**^: the formation/development of the ducts, followed by their separation; fusion of the lower portions to form the uterus, cervix, and upper vagina; and reabsorption of the uterine septum, creating a single uterine cavity. Müllerian anomalies occur due to failure in any of those stages and can therefore be anomalies of development, fusion or reabsorption, with various manifestations, including a unicornuate, bicornuate, or septate uterus^**(^9^)**^. Fusion and reabsorption anomalies can also give rise to a longitudinal or transverse vaginal septum^**(^2^,^10^)**^.

## IMAGING METHODS

Hysterosalpingography, which has been used for almost a century, is the oldest method of evaluating uterine malformations. Although it allows examination of the uterine cavity, cervical canal, and tubal patency, it does not reveal the external contour of the uterus or obstructing anomalies such as a non-communicating uterine horn; nor can it identify extrauterine alterations, including those affecting the ovaries or urinary tract^**(^8^)**^. Two-dimensional ultrasonography, which is widely available and affordable, has good (90-92%) sensitivity for detecting uterine anomalies and is an effective screening tool^**(^11^)**^; it can be used in order to identify cases of uterine agenesis and cavity duplication, provided that well-defined imaging criteria are followed to reduce interobserver variability^**(^12^)**^.

Three-dimensional ultrasound and magnetic resonance imaging (MRI) both allow volumetric acquisitions and generate images in any plane, enabling detailed analysis of the uterine cavity and its external contour, regardless of the position of the uterus in the pelvis. For diagnosing müllerian anomalies, MRI is considered the gold standard, offering greater operator independence and ease in identifying other, concomitant anomalies. On MRI, it is possible to characterize the uterine contour, tubal ostia, and cervical os, which allows an accurate diagnosis to be made^**(^13^)**^. For complex cases, as well as for cases of patients with accompanying malformations, deep endometriosis, or a history of surgery or trauma, MRI is indicated^**(^2^)**^.

## HISTORY OF THE CLASSIFICATION OF MÜLLERIAN ANOMALIES

**Figure 1:**
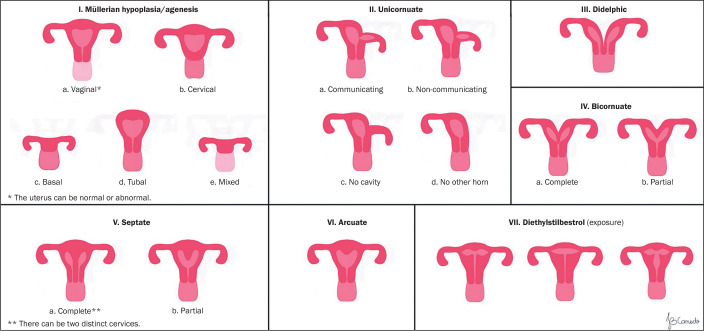
Schematic representation of the 1988 AFS classification of müllerian anomalies.

The first classification of müllerian anomalies was proposed by a pathologist named Eduard Kaufmann and published by the obstetrician Julius Jarcho in 1946^**(^14^)**^, establishing an initial milestone for the diagnosis of these conditions. Fast forward to 1988, and the AFS published its comprehensive system, which structured the anomalies into seven distinct classes, depending on the degree of development and fusion of the müllerian ducts (Figure 1). These classes included everything from müllerian agenesis to uteri exposed to diethylstilbestrol.

### 1988 AFS classification

**Class I:** Agenesis or hypoplasia, with specific subdivisions for vaginal, cervical, basal, tubal, or combined alterations.

**Class II:** Unicornuate uterus, with variants ranging from a rudimentary communicating horn to the complete absence of a contralateral horn.

**Classes III to VII:** Ranging from didelphic to bicornuate, septate, and arcuate uteri, to exposure to teratogenic substances such as diethylstilbestrol.

The 1988 AFS classification was valued for its simplicity and effectiveness in correlating anatomical forms with clinical prognoses for pregnancy outcomes^**(^15^)**^. However, because it was one of the first initiatives to systematize the classification of müllerian anomalies, the use of the AFS classification had some limitations, mainly the subjectivity inherent in the lack of well-defined diagnostic criteria; the difficulty in categorizing anomalies involving the vagina and cervix; and the fact that complex anomalies were allowed to be classified in an individualized manner, those classifications therefore being more dependent on the heterogeneous knowledge regarding the entity and the different lexicons of the professionals involved^**(^6^)**^.

### 2013 ESHRE/ESGE classification

In response to the limitations of the AFS classification, the ESHRE/ESGE introduced a more detailed system in 2013 that not only addressed uterine anomalies but also included specific categories for cervical and vaginal anomalies, with the aim of eliminating the subjective diagnosis of the original AFS classification, as well as allowing differentiation between a septate uterus and other, similar conditions, regardless of the absolute morphometric criteria.

**Figure 2:**
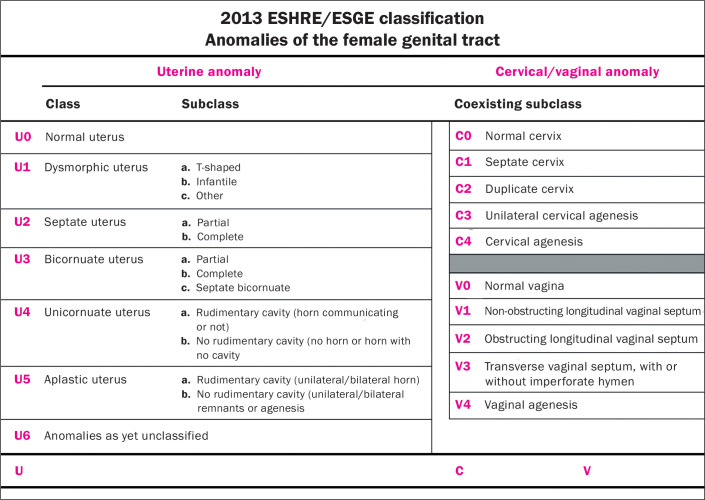
Scheme available in the 2013 ESHRE/ESGE classification.

**Figure 3:**
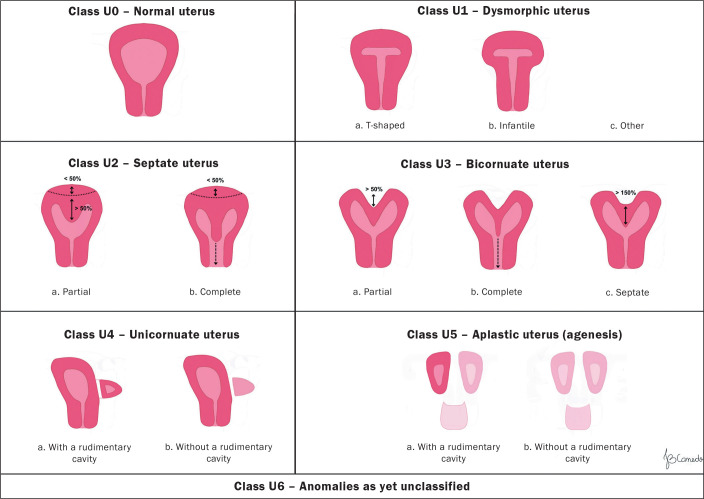
Schematic representation of the 2013 ESHRE/ESGE classification of uterine anomalies.

The ESHRE/ESGE classification is based on the pelvic anatomy and divides the main classes according to anatomical alterations derived from the same embryological origin, whereas the subclasses are divided on the basis of anatomical variations of the main classes. Cervical and vaginal anomalies are divided into supplementary and independent subclasses^**(^5^)**^, as illustrated in Figures 2 and 3.

In the ESHRE/ESGE classification, class U0 includes cases with a normal uterus, defined by a straight or curved interostial line, with small fundal myometrial invagination that does not exceed 50% of the uterine wall thickness. This classification avoids the use of absolute numbers, because the authors believed that uterine dimensions and uterine wall thickness can vary between patients. Therefore, they defined uterine deformity based on uterine anatomical proportions, such as uterine wall thickness.

Class U1, or dysmorphic uterus, includes cases with a normal external uterine contour but with an abnormal shape of the uterine cavity. Its subclasses are as follows: U1a (T-shaped uterus), characterized by a narrow uterine cavity due to thickening of the lateral walls, with the majority corresponding to the uterine body and a smaller portion corresponding to the cervix; U1b (infantile uterus), characterized by a narrow uterine cavity without thickening of the lateral walls, with the majority corresponding to the cervix and a smaller portion corresponding to the uterine body; and U1c, or “other”, which includes minor deformities of the uterine cavity, including those with small invagination of the basal myometrium, which represents less than 50% of the thickness of the uterine wall.

Class U2, also known as a septate uterus, is an anomaly in which the uterine cavity presents myometrial or fibrous invagination from the uterine fundus, currently described as a septum, which exceeds 50% of the thickness of the uterine wall. It is divided into two subclasses, depending on the degree of deformity of the uterine body: U2a, or a partial septate uterus, characterized by the presence of a septum that partially divides the uterine cavity, above the level of the internal cervical os; and U2b, or a complete septate uterus, characterized by the presence of a septum that completely divides the uterine cavity up to the level of the internal cervical os. Patients with a complete septate uterus (a class U2b anomaly) might present cervical anomalies (such as a uterus with a septate cervix), with or without vaginal defects.

Class U3, also known as a bicornuate uterus, presents as an abnormal contour of the uterine fundus, with an indentation of the serosa in the midline that exceeds 50% of the thickness of the uterine wall. The indentation can partially or completely divide the uterine body, in some cases including the cervix, vagina, or both. Class U3 is divided into three subclasses: U3a (a partial bicornuate uterus), characterized by an indentation of the serosa that partially divides the uterine body above the level of the cervix; U3b (a complete bicornuate uterus), characterized by an indentation of the serosa that completely divides the uterine body up to the level of the cervix; U3c (a septate-bicornuate uterus), characterized by an additional absorption defect in which the thickness (depth) of the indentation of the uterine fundus exceeds 150% of the thickness of the uterine wall.

Class U4 (a unicornuate uterus) is an anomaly in which there is unilateral development of the uterus, with the contralateral portion being incompletely formed or absent. It is divided into two subclasses: U4a, characterized by a unicornuate uterus with a functional rudimentary cavity, which has a functional contralateral horn that can be communicating or non-communicating; and U4b, characterized by a unicornuate uterus without a functional rudimentary cavity, with a non-functioning contralateral uterine horn or with agenesis of this contralateral portion.

Class U5 (an aplastic uterus) is defined by the absence of any fully developed or unilateral uterine cavity. Aplastic uteri are divided into two subclasses: U5a, those with a rudimentary (functional) cavity, characterized by the presence of a functional horn (bilateral or unilateral); and U5b, those without a rudimentary (functional) cavity, with uterine remnants or complete agenesis of the uterus.

Finally, class U6 is reserved for cases not yet classified.

The ESHRE/ESGE classification also categorizes coexisting cervical and vaginal anomalies on a scale from 0 to 4 (Figure 2). The authors removed the term “arcuate uterus” from the classification, because it was considered confusing and because it was pointed out that there was a need for clearer definitions. Therefore, it was decided that the septate uterus classification should include only patients with midline invagination of the basal myometrium that occupied 50% of the uterine wall thickness. A new subcategory under the general term “other” was added to class U1 (dysmorphic uterus), giving the opportunity to include all minor deformities of the endometrial cavity, including midline invaginations of the basal myometrium occupying 50% of the uterine wall thickness, making it clear that further clinical research would be required in order to determine the clinical value of this variant^**(^5^)**^.

Chief among the limitations of using the ESHRE/ESGE classification is the complexity of its clinical applicability, because it depends on individual analysis and the interpretation of anomalies can therefore vary among professionals. Ludwin et al.^**(^15^)**^ compared the ESHRE/ESGE (European) classification with the AFS (American) classification and observed that the application of the ESHRE/ESGE criteria can result in the overdiagnosis of septate uterus, with a significant, nearly threefold, increase in the frequency of its recognition, probably related to the cutoff point used, leading to excessive and unnecessary treatments^**(^15^)**^.

The 1988 AFS and 2013 ESHRE/ESGE classifications differ mainly in objectivity and detail.

The 1988 AFS classification is based on general anatomical descriptions and has been criticized for allowing subjectivity in the interpretation of müllerian anomalies. The AFS classification is more conservative and focused on specific uterine anomalies, whereas the ESHRE/ESGE classification introduced clearer, more standardized criteria and broadened the scope to include other malformations of the genital tract, but at the potential cost of overdiagnosis and unnecessary interventions.

### Comparison between the 1988 AFS and 2021 ASRM classifications

The wide range of müllerian anomalies, combined with the rarity of these conditions and the absence of universal objective criteria, continues to complicate their identification and treatment^**(^6^)**^. An ideal classification would facilitate the identification of these anomalies, improve communication between health professionals, and consequently optimize the clinical care provided to affected women. In this context, the need to update and refine existing classifications led to the creation of a new classification by the ASRM in 2021.

The ASRM convened a multidisciplinary group, including members of the ASRM itself, the Society of Reproductive Surgeons, and the American Society of Pediatric and Adolescent Gynecology, as well as radiologists specializing in the imaging of müllerian anomalies. The group conducted a comprehensive analysis of the existing classifications, highlighting their merits and deficiencies.

The multidisciplinary ASRM group identified the 1988 AFS classification as the most practical and widely accepted because of its simplicity and visual clarity^**(^4^)**^. Despite its limited scope-it did not include all forms of anomalies, nor did it include cervical and vaginal abnormalities, which were described in addition to the predominant malformation of the uterus-the AFS classification was adopted as the basis for the new system That was considered preferable to the development of a completely novel system.

**Figure 4:**
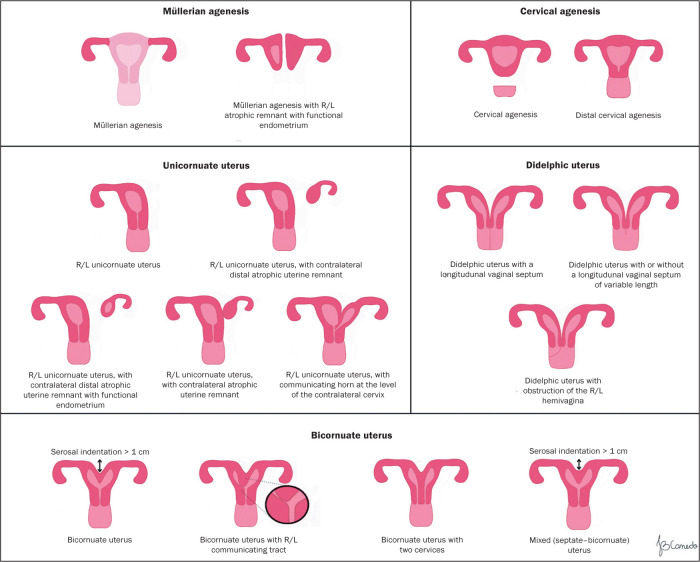
Schematic representation of the 2021 ASRM classification.

**Figure 5:**
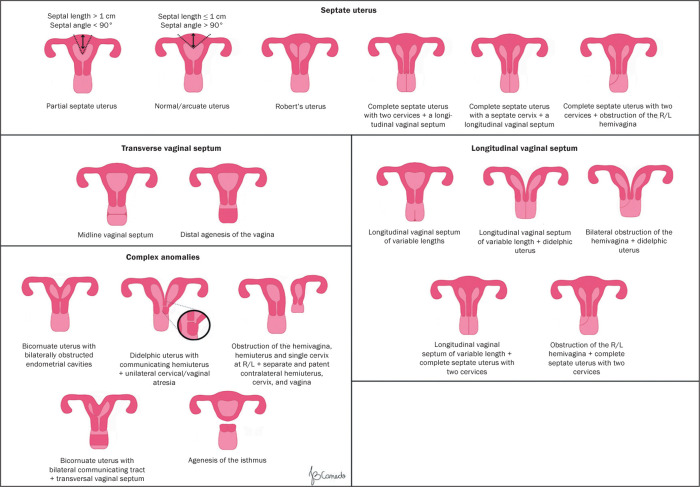
Schematic representation of the 2021 ASRM classification.

In the 2021 ASRM classification, the AFS categories have been expanded to include three new classes: longitudinal vaginal septum, transverse vaginal septum, and complex anomalies. The illustrations have been modernized to maintain ease of recognition, while more precise diagnostic criteria have been established to distinguish, in particular, between bicornuate and septate uteri (Figures 4 and 5, respectively). A consistent, understandable lexicon has also been established to facilitate communication. Different than in the AFS classification, the anomaly categories are no longer numbered but are identified by descriptive terminology.

The 2021 ASRM classification is divided into nine main categories:

• Müllerian agenesis (with or without unilateral or bilateral atrophic uterine remnants and with or without a functioning endometrium)
• Cervical agenesis
• Unicornuate uterus (with or without a contralateral rudimentary hemiuterus and with or without a functioning endometrium)
• Didelphic uterus (two hemiuteri and a non-fused or duplicated cervix)
• Bicornuate uterus (partially fused bodies due to indentation of the serosa)
• Septate uterus
• Longitudinal vaginal septum
• Transverse vaginal septum
• Complex anomalies

**Figure 6:**
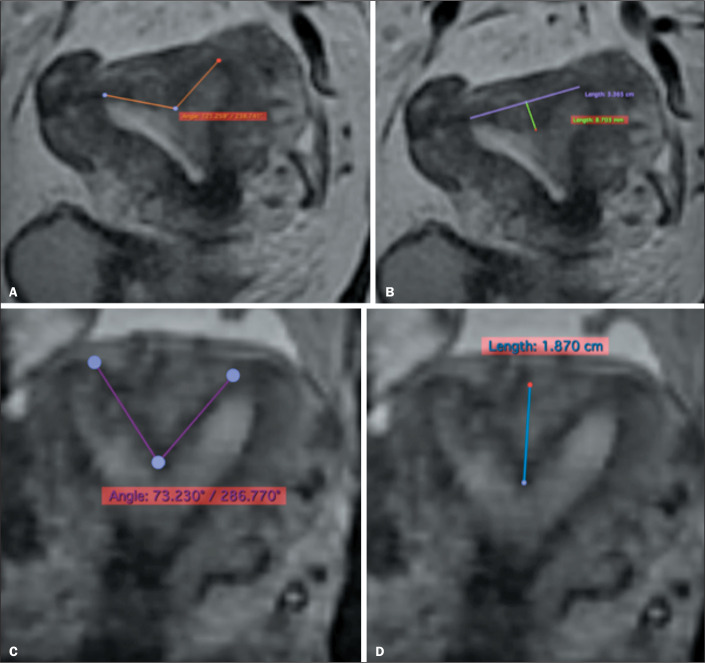
T2-weighted, volumetric, turbo spin-echo MRI scans showing how to make a correct measurements of the myometrial or fibrous invagination of the uterine fundus, by angle (A,C) and by distance (B,D-length in centimeters). The diagnoses were arcuate uterus (A,B) and partial septate uterus (C,D).

In the 2021 ASRM classification, specific criteria have been defined to categorize septate, arcuate, and bicornuate uteri. In comparison with the 2016 ASRM guidelines^**(^7^)**^, modifications were made for the septate uterus, now defined as a uterus with an endometrial septum (myometrial or fibrous invagination from the uterine fundus) that is more than 1 cm long, angled at less than 90°, and has a normal external contour of the uterine fundus (Figure 6). A septate uterus may also present septation of the cervix, in which case it is referred to as a complete septate uterus, characterized by a continuous or discontinuous division with the myometrial or fibrous uterine septum, without circumferential stroma^**(^6^)**^.

Kauffman considered the arcuate uterus to be a variation of the bicornuate uterus^**(^14^)**^, rather than a differential diagnosis within the septate uterus spectrum^**(^16^)**^. However, it was mentioned at the time as a possible variation of normal, with undetermined clinical repercussions. After the term “arcuate uterus” was omitted from the 2013 ESHRE/ESGE classification, it reappeared in the 2021 ASRM classification, defined as a uterus with a myometrial septum of less than 1 cm, maintaining the normal external contour of the uterine fundus. The authors stated that an arcuate uterus is a clinically insignificant finding and is therefore considered a variant of normality.

In the 2021 ASRM classification, the bicornuate uterus is defined as an indentation in the serosa (of the external contour) greater than 1 cm that can be accompanied by duplication of the cervix, which may be partially fused (with stroma separating the cervical canals) or with two completely separate cervices (with independent stroma and myometrium). Some uteri may present adenomyosis or more vascular or connective structures between the horns than myometrium, which in some rare cases creates uncertainty due to the adoption of a single criterion of the serosa to differentiate septate from bicornuate. Connective structures refer to connective tissues that can be present between the uterine horns, contributing to the morphology of the uterine cavity. Connective tissue is composed of an extracellular matrix rich in collagen and other structural proteins, in addition to cells such as fibroblasts and myofibroblasts. In the context of differentiating between a septate and bicornuate uterus, the predominance of connective tissue in the uterine fundus can make classification difficult; if the area between the horns presents a predominance of connective tissue and reduced vascularization, it can resemble a fibrous septum.

**Figure 7:**
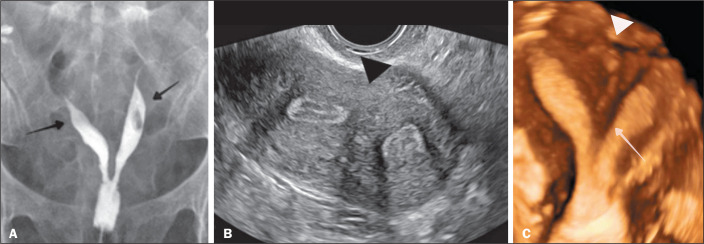
Hysterosalpingography (A) showing two uterine cavities (arrows). Ultrasound (B) and three-dimensional ultrasound (C) showing a normal external uterine contour (arrowhead) and a septum with a depth greater than 1.0 cm and an angle of less than 90°, with a septum that does not extend beyond the internal cervical os (arrow). According to the 1988 AFS (original American) classification, this would be classified as a partially septate uterus (subclass Vb). According to the 2013 ESHRE/ESGE (European) classification, this would be a partially septate uterus (subclass U2a), characterized by the presence of a septum partially dividing the uterine cavity above the level of the internal cervical os, with a normal (class C0) cervix and a normal (class V0) vagina.

The 2021 ASRM classification also allows cross-comparison between categories. That can aid in the diagnosis and choice among therapeutic options for complex or controversial cases (Figure 7). With these changes, the 2021 ASRM classification serves the purpose of building on the simplicity, strengths, and easy identification of the 1988 AFS classification, while expanding and updating the system to include cervical and vaginal anomalies. It incorporates valuable ideological pillars, notably the choice of description rather than numbering, which allows for more accurate and intuitive identification of anomalies. In addition, it is designed to encompass complex anomalies and different anatomical structures, which helps avoid underdiagnosis, especially at centers that are less specialized^**(^6^)**^.

In order to facilitate the dissemination and understanding of the 2021 classification, the ASRM provides a highly complete and practical online tool for the differential diagnosis of anomalies, which can be easily consulted on the ASRM website. This interactive tool presents, on the main page for each class, the descriptors that facilitate the identification of the malformation. In addition, it includes many possible variations of each class, making the classification and diagnosis process more accessible and detailed. The practicality of this online tool allows health care professionals to navigate intuitively, with standardized information that simplifies the analysis and promotes diagnostic accuracy. Thus, the 2021 ASRM classification also aims to raise awareness about the diversity of müllerian anomalies, standardizing terminology to facilitate communication between professionals and support research in scientific databases, improving the quality of records. Furthermore, it acts as an educational tool, providing online information on the presentation, diagnosis, and treatment of anomalies, being applicable for professionals of all levels, including students and residents, and promoting knowledge and the defense of patient rights.

## DISCUSSION AND FUTURE DIRECTIONS

**Figure 8:**
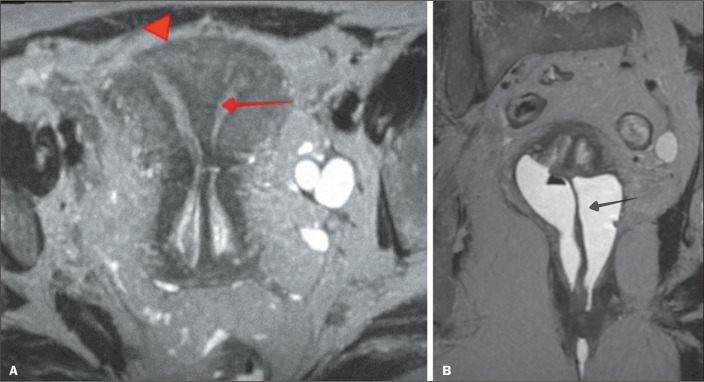
T2-weighted turbo spin-echo MRI scans, in the axial and coronal planes (A and B, respectively). Note the normal external uterine contour (arrowhead) and the septum with a depth greater than 1.0 cm and a septal angle of less than 90° (arrow), together with a septum that extends beyond the internal cervical os and a longitudinal vaginal septum (arrow in B). According to the 1988 AFS (original American) classification, it would be classified as a complete septate uterus (subclass Va). According to the 2013 ESHRE/ESGE (European) classification, it would be classified as a complete septate uterus (subclass U2b), with a duplicated (class C2) cervix and a non-obstructing longitudinal (class V1) vaginal septum. According to the 2021 ASRM (latest American) classification, it would be classified as a complete septate uterus with a duplicated cervix and longitudinal vaginal septum.

Despite the creation of well-defined diagnostic criteria, some authors argue that the cutoff values established in the 2021 ASRM classification appear to be arbitrary and not supported by robust scientific evidence, mainly in relation to the definitions of the most common anomalies, including arcuate, septate, and bicornuate uteri^**(^16^)**^. Under certain circumstances, there can even be discrepancies between these criteria, resulting in a greater number of inconclusive diagnoses. For example, the classification does not cover cases in which the uterus presents an internal indentation angle of less than 90°, but with a depth of less than 10 mm; similarly, an indentation depth greater than 10 mm accompanied by a wide angle places the situation once again in a gray area between the definitions of arcuate uterus and septate uterus (Figure 8). Another limitation consists of the reaffirmation of the broad spectrum of müllerian anomalies, suggesting that the current categories may not encompass all existing variants, which indicates the need for future revisions and updates to accommodate new discoveries, to better understand them, and, consequently, to better treat them.

One important contribution to the evolution of classifications over the years, maintained in the most recent classification, is the concept of continuity in development. It also reflects something that is increasingly observed today, due to advances in knowledge and imaging techniques, which is “noncompliance” with the rule of development in the caudocranial direction (fusion and reabsorption of structures arising from the müllerian ducts), which might have resulted in underdiagnoses, especially in relation to cervical and vaginal fusion anomalies.

Most of the classes in the 2021 ASRM classification remain linked to the final anatomical structures of the genitourinary tract as the main criterion. Perhaps the embryological origin of the structures could be a better reference, so that different structures could be in the same class. In cases reported in the current literature, associated genitourinary anomalies are only partially evaluated, largely because of the diagnostic approach or flow.

Use of the term “T-shaped uterus” led to underdiagnosis and was heavily criticized in the European classification. A suggested alternative was the term “congenital”, which is more comprehensive and thus allows the inclusion of possible new diagnoses to be described in the literature. Likewise, the discussion on “accessory cavitated uterine malformations” gained more attention in the literature. This is a new approach to findings that can resemble focal adenomyosis but are somewhat outside the scope of this review.

**Figure 9:**
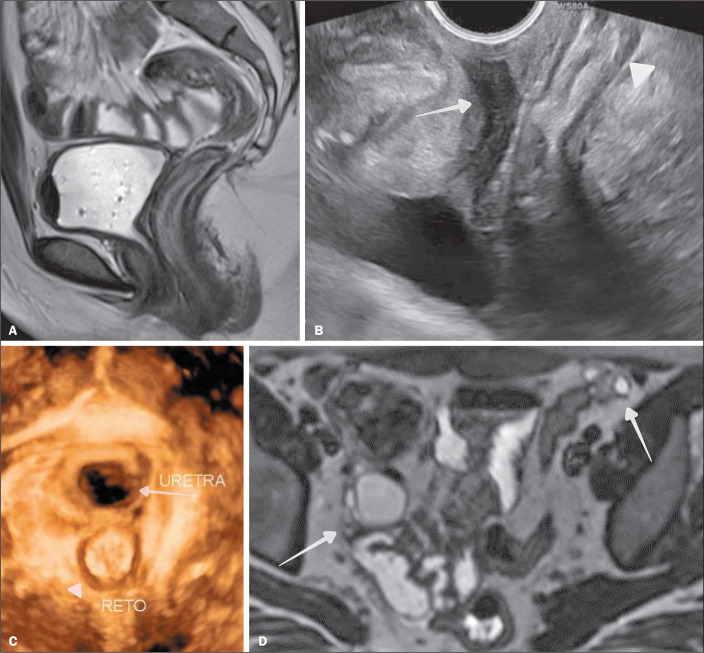
Mayer-Rokitansky-Küster-Hauser syndrome. Sagittal T2-weighted turbo spin-echo MRI (A), transvaginal ultrasound (B), and three-dimensional pelvic floor ultrasound (C), demonstrating agenesis of the uterus, cervix, and vagina. Urethra (arrow in B) with a larger than usual caliber, where the gel was introduced. The arrowhead in B shows the rectum. D: Axial T2-weighted MRI showing ovaries with a normal appearance (arrows). These findings were associated with right-sided renal agenesis (image not available). According to the 1988 AFS (original American) classification, this case would be categorized as combined agenesis (subclass Ie). According to the 2013 ESHRE/ESGE (European) classification, it would be categorized as uterine agenesis without a rudimentary horn (subclass U5b), with cervical and vaginal agenesis (classes C4 and V4, respectively). According to the 2021 ASRM (latest American) classification, it would be classified simply as müllerian agenesis.

The 2021 ASRM classification maintains an easy-to-understand format and offers standardized terminology (Figure 9). With its common, accessible language, this classification is expected to facilitate the execution of clinical studies, which are essential for the advancement of reproductive health research.

## CONCLUSION

The evolution of the classification of müllerian anomalies reflects an ongoing effort to improve the diagnostic accuracy and clinical management of these complex conditions. The transition from the AFS classification to the European classification to the more recent ASRM classification, exemplifies the progress in our understanding of the anatomical variations of the female reproductive tract and the need for more detailed and less subjective diagnostic approaches. Radiologists performing investigational examinations for uterine malformations must be aware of the benefits of each classification in order to appropriately classify the findings, understanding that there are variables in the classifications and that it is necessary to facilitate the diagnosis in order to guide the referring physician in finding the best course of action to follow.

The 2021 ASRM classification not only expanded diagnostic categories but also facilitated a clearer common language for health care professionals. This advancement is vital to improving reproductive outcomes and quality of life for patients affected by these anomalies. Finally, the flexibility of the new classification to incorporate future discoveries highlights the importance of ongoing review, ensuring that the classification of müllerian anomalies remains relevant and applicable to scientific and clinical advances.
